# Multidisciplinary management of ankyloglossia in childhood. Treatment of 101 cases. A protocol

**DOI:** 10.4317/medoral.20736

**Published:** 2015-11-22

**Authors:** Elvira Ferrés-Amat, Tomasa Pastor-Vera, Eduard Ferrés-Amat, Javier Mareque-Bueno, Jordi Prats-Armengol, Eduard Ferrés-Padró

**Affiliations:** 1 DDS, PhD. Service of Oral and Maxillofacial Surgery. Hospital de Nens de Barcelona. Service of Pediatric Dentistry. Hospital de Nens de Barcelona. Barcelona. Spain. Department of Oral and Maxillofacial Surgery, Faculty of Dentistry, Universitat Internacional de Catalunya. Barcelona, Spain; 2 Psy D, PhD. Head of the Service of Speech and Orofacial Myofunctional Therapy. Hospital de Nens de Barcelona. Barcelona. Spain; 3 DDS, MSc, PhD St. Service of Oral and Maxillofacial Surgery. Hospital de Nens de Barcelona. Barcelona. Spain. Department of Oral and Maxillofacial Medicine and Oral Public Health, Faculty of Dentistry, Universitat Internacional de Catalunya, Barcelona, Spain; 4 MD, DDS, FEBOMS, PhD. Service of Oral and Maxillofacial Surgery. Hospital de Nens de Barcelona. Barcelona. Spain. Department of Oral and Maxillofacial Medicine and Oral Public Health, Faculty of Dentistry, Universitat Internacional de Catalunya, Barcelona, Spain; 5 MD, DDS. Service of Oral and Maxillofacial Surgery. Hospital de Nens de Barcelona. Barcelona. Spain. Department of Oral and Maxillofacial Surgery. Faculty of Dentistry, Universitat Internacional de Catalunya. Barcelona. Spain; 6 MD, DMD, OMS, PhD. Head of the Service of Oral and Maxillofacial Surgery. Hospital de Nens de Barcelona. Barcelona. Spain

## Abstract

**Background:**

Partial ankyloglossia is a limitation which restricts the possibility of protrusion and elevation of the tip of the tongue due to the shortness of either the lingual frenulum or the genioglossus muscles or both. The principal objective of this paper is to present our protocol of action for the treatment of ankyloglossia. The specific objectives are to study patients with ankyloglossia treated by the Service of Maxillofacial Surgery and the Service of Speech Therapy of our pediatric Hospital, describe the diagnostic procedures, the pre-surgical intervention, the surgical technique undertaken and the post-surgical rehabilitation taking into account the level of collaboration of the patients, and finally, describe the surgical complications and the referral of patients.

**Material and Methods:**

This is a descriptive study of healthy patients, without any diagnosis of syndrome, ranging between 4 and 14 years that have been surgically treated and rehabilitated post-surgery within a period of 2 years.

**Results:**

101 frenectomies and lingual plasties have been performed and patients have been treated following the protocol of action that we hereby present. After the surgical intervention, the degree of ankyloglossia has been improved, considering correction in 29 (28%) of the patients (95% CI: 20%, 38%), reaching, with the post-surgical orofacial rehabilitation, a correction of 97 (96%) of the participants (95% CI: 90%, 98%).

**Conclusions:**

The chosen surgical technique for moderate-severe ankyloglossia in our centre is the frenectomy and lingual plasty. The myofunctional training begins one week before the surgical intervention so that the patients learn the exercises without pain.

**Key words:**Ankyloglossia, tongue-tie, lingual frenum.

## Introduction

Ankyloglossia (partial) is defined as a limitation of the possibilities of the protrusion and elevation of the tip of the tongue due to either the shortness of the frenulum or the genioglossus muscles or both. (1,2) It seems to have a genetic aetiology and normally occurs in children between 1 and 3 years old. Its diagnosis is clinical-functional. It is considered that there is a hypertrophic lingual frenulum when the tongue mobility is reduced. (3,4)

Ankyloglossia can be observed at different ages with specific indications for treatment for each group. (4) Our centre differentiates ankyloglossia in neonates and infants from that occurring during childhood and adolescence.

The limitation in lingual mobility in neonates could cause three alterations in breast feeding: soreness, cracked nipples or mastitis of the mother, poor weight gain of the newborn, excessively long period of time in each breast feed. (5-8)

The restriction of lingual mobility during childhood and adolescence can cause alterations in bone growth of either the orofacial structures or the oral functions of the child or both. (9-12)

With regard to phonetic sound articulation, the sounds which more often alter is the /s/, producing a distorted sound, as well as the multiple /r/ sound, being substituted by other sounds or not producing vibration. The first case is explained by the position of a lowered tongue, and in the second case due to the restriction of lingual mobility thus impeding the tongue to produce a total closure against the palate for the creation of the necessary vibration to emit the sound correctly. There are other sounds that can be altered as a result of ankyloglossia, like the /t/, /d/ and /l/ but these occur less frequently. (10)

As regards swallowing, ankyloglossia causes atypical deglutition due to insufficient palatal support to produce a mature adult swallow. The physiological process of the child which goes from the childhood swallow to the adult one in the early years of life is interrupted due to the restricted driving force in the infant with hypertrophic lingual frenulum. (9,12)

With respect to growth of the stomatognathic system, the tongue, jaws, among others, plays the function of shaping the palate essential for proper bone growth. (12) Ankyloglossia impedes lingual elevation, hence this may cause a narrowing of the upper maxillary due to the lack of transversal growth and provoking a cross-bite. In other cases, it will generate an abnormal growth of either the mandible or an anterior open bite or both as a result of the low tongue position. In some patients, a short and hypertrophic lingual frenulum will cause a diastema between the lower central incisors and could provoke difficulties in orthodontic treatments with removable appliances. A bilateral open bite can also be produced when the tongue thrusts between the maxillaries to carry out its usual functions or is at rest. This fact comes usually associated with other factors such as a masticating musculature with a weak muscle tone or macroglossia. (9,11,12)

The alterations that reduced tongue mobility could cause require the need for a description or classification of the varying degrees of ankyloglossia and a protocol of action, (13-15) on the one hand to unify diagnostic criteria among the different professionals which treat this problem, and on the other hand, to define the seriousness of the problem when it is presented to us and thus reach a common set of therapeutic criteria. (14-16)

The purpose of this present article is to present the protocol of action of the Fundació Hospital de Nens de Barcelona (FHNB) for the treatment of ankyloglossia in childhood and adolescence. The specific objectives are to study patients with ankyloglossia during childhood treated jointly by the Maxillofacial Surgery Service and Speech Therapy Service and Orofacial Rehabilitation of the FHNB, describe the diagnostic procedures, the pre-surgical action, the surgical technique undertaken and the post-surgical rehabilitation, taking into account the level of collaboration of the patients, and finally reporting the surgical complications and patient referral.

## Material and Methods

It is a cohort study. This is a transversal descriptive study of patients between 4 and 14 years that have been diagnosed with ankyloglossia and surgically treated by means of a frenectomy and lingual plasty by the Maxillofacial Surgery Service and the Speech Therapy and Orofacial Rehabilitation Service within a period of 2 years (from September 2012 to September 2014).

This investigation project was presented to the Scientific Council of the Fundación Hospital de Nens de Barcelona. The mentioned Committee analysed all the presented documentation, and accordingly, unanimously, emitted a favorable report from the ethical point of view of the investigation, seeing that in the mentioned observational study none modification of the standard assistance protocol of our hospital is done, respecting completely the participant’s anonymity and in any way at all their identity can be known, as well as their addition to the study is a voluntary act.

The selection of the patients has been based on the following criteria of inclusion and of exclusion.

Selection criteria of the sample included healthy patients, without any diagnosis of syndrome.

The criteria for exclusion: the patients that did not sign the informed consent to be part of the study, the patients that have not attended the postoperative medical check or even those patients that have not undertaken all the indicated rehabilitation sessions according to our protocol.

We recorded the sex and age of the patients in a data collection sheet, the degree of ankyloglossia at the beginning, pre surgical action, the surgical technique carried out, the degree of post-surgical ankyloglossia and subsequently post-surgical rehabilitation. The surgical complications and the degree of collaboration of the patients during the rehabilitation sessions were recorded and also who carried out the referral of aforementioned patients.

**Figure 1:**
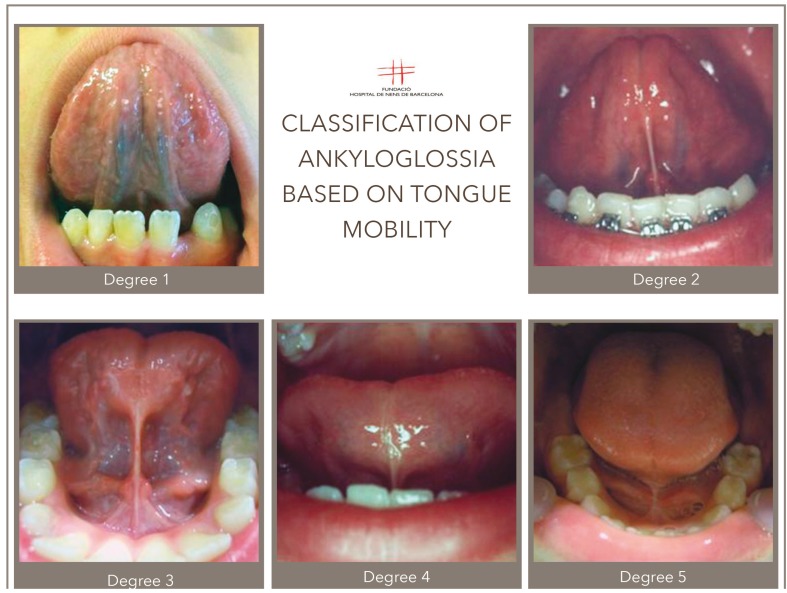
Degrees of Ankyloglossia. Classification.

Data is collected using a classification of ankyloglossia based on the degree of limitation of lingual mobility (Fig. 1). It is considered that hypertrophic lingual frenulum exists when lingual mobility is reduced, and in response to that limitation, we defined 5 degrees:

Degree 1: Conceptual definition: This is a tongue having totally free movement; the tongue tip can reach its highest point. Operative definition: The patient is requested to lift the tip of the tongue towards the palate with the mouth open, and this reaches its maximum point on touching the palate. The tongue reaches a perfect degree of verticality.

Degree 2: Conceptual definition: A mild frenulum exists, although the tongue has almost got full mobility. It is observed when the mouth is opened at maximum; there is a slight impediment in tongue elevation. Operative definition: The patient is requested to lift the tongue, and this is reached to three quarters of the intraoral space, but touching the palate is not achieved.

Degree 3: Conceptual definition: Moderate hypertrophy is recorded with a moderate lingual mobility impairment. Operative definition: On requesting the patient to lift the tongue during the medical examination, it is observed that the tongue occupies half of the intraoral space, causing the appearance of a forked tongue or in the shape of a heart due to the tension produced as a result of restricted lingual mobility.

Degree 4: Conceptual definition: It is a frenulum with a rather reduced level of lingual mobility; the tongue is low but the base of the tongue and frenulum can still be observed. The degree is severe and consequently requires surgery. Operative definition: The patient is requested to lift the tongue and only a quarter of the intraoral space is reached; it is a tongue with reduced movements and hence, bone growth and oral functions are impeded.

Degree 5: Conceptual definition: Lingual mobility is totally restricted. It is what is termed serious ankyloglossia. The sublingual frenulum practically impedes the very movement of the tongue essential for the optimum growth of the orofacial functions, Therefore, surgery is required here too. Operative definition: During the examination, the base of either the tongue or the frenulum cannot be onserved, due to the extreme limitation of the frenulum at degree 5 level. This limitation of lingual movements makes it impossible for the normal development of the stomatognathic system structures, thus totally affecting their functions.

The frenulum is assessed as hypertrophic when the degree is 4 or 5 and a normal frenulum when it is below degree 3. We consider that frenulums 4 or 5 require surgery due to the weak driving power. Lingual frenulum 3 is evaluated as requiring surgery if it related to another alteration, pathology or disorder.

**Figure 2:**
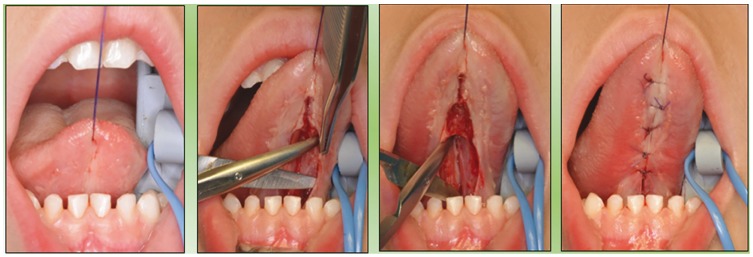
Surgical Technique. Frenectomy, rhomboid plasty and miotomy.

The surgical treatment of ankyloglossia is performed under local anaesthesia and intravenous sedation. The surgical technique used in all the cases is the frenectomy and rhomboidal plasty: the submucous infiltration is carried out with an anaesthetic solution with a vasoconstrictor (articaine 4% with epinephrine 1:100.000), a horizontal-rhomboidal incision is made, a sub mucous dissection (5 mm) of the margins and the dissection of the two genioglossus muscles, various myotomies were performed, at different levels, in both muscles, after a careful haemostasis, it was closed with simple stitches of 4/0 (polyglycolic acid) and finally, the transection of the mandibular attachment of the fibres (Fig. 2).

Orofacial rehabilitation, in our protocol, begins one week before surgery; it is an action which differentiates us from other protocolised actions. The motive is justified by the fact that the child learns to perform the praxis of the treatment without pain.

**Figure 3:**
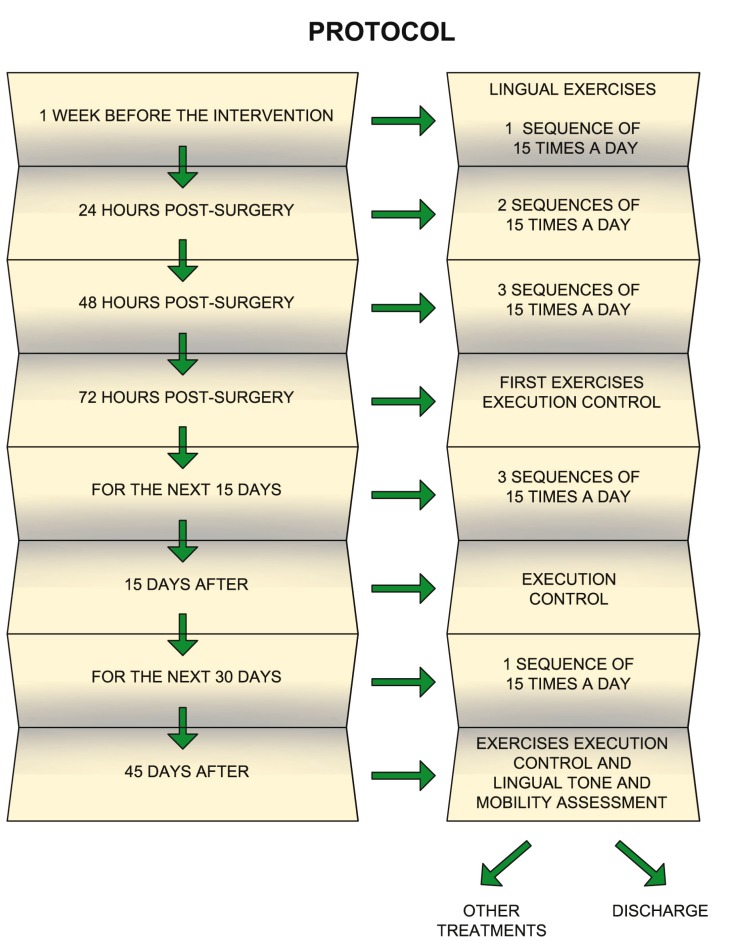
Protocol of Ankyloglossia rehabilitation.

After surgery, the praxis is repeated 24 hours after the intervention, (2 sequences of 15 times) and 48 hours after surgery, the praxis is repeated 3 times a day (Fig. 3).

As regards post-surgical check-ups, they were done at 72 hours, at 15 days, and at 45 days, to evaluate the performance of the praxis, the tone and motor development of the lingual musculature and the suppleness of the scar tissue. Moreover, the state of the phonetic articulation and the oral functions are also evaluated.

On many occasions, the rehabilitation of the tongue and the dyslalia as a result of ankyloglossia are resolved parallelly. This fact is explained through the free movement of the tongue and the work of the lingual muscles.

Once this period of time has passed, the patient is discharged from post-frenectomy rehabilitation and if necessary, other speech therapy treatments are initiated.

The limitation of this study is the lack of control group of operation alone without rehabilitation, although postoperative rehabilitation is preferable.

Sample size - Data comes from a still ongoing cohort study and therefore, no previous size calculus has been done for these specific results. Nevertheless, a sample size (n=101) is powerful enough to estimate a proportion of around 0.96 with a 95% confidence interval (CI) with a precision of ± 0.04.

Analysis - Proportion confidence intervals were calculated based on binomial distribution. We used chi-square/Fisher tests for categorical data and Student t-tests for independent samples for continuous data. Data was analysed with R: A language and environment for statistical computing, version 3.1.2.

## Results

During the period of the study, 101 patients with ankyloglossia underwent treatment (38 girls and 63 boys) ranking in age from 4 to 14 years old. The characteristics of the patients are shown in  Table 1, Table 2.

**Table 1:**
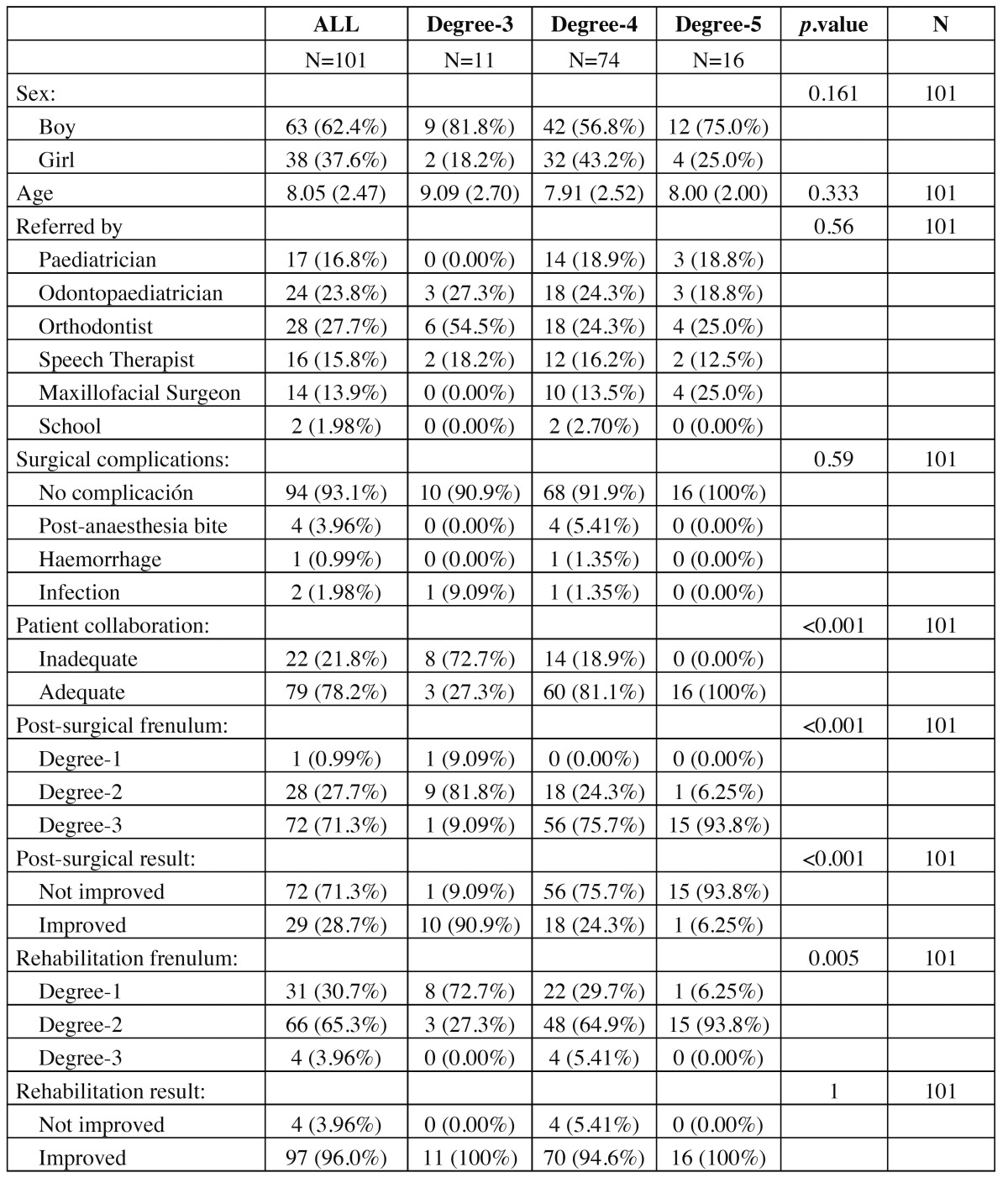
General description of the participants according to the degree of initial Ankyloglossia.

**Table 2:**
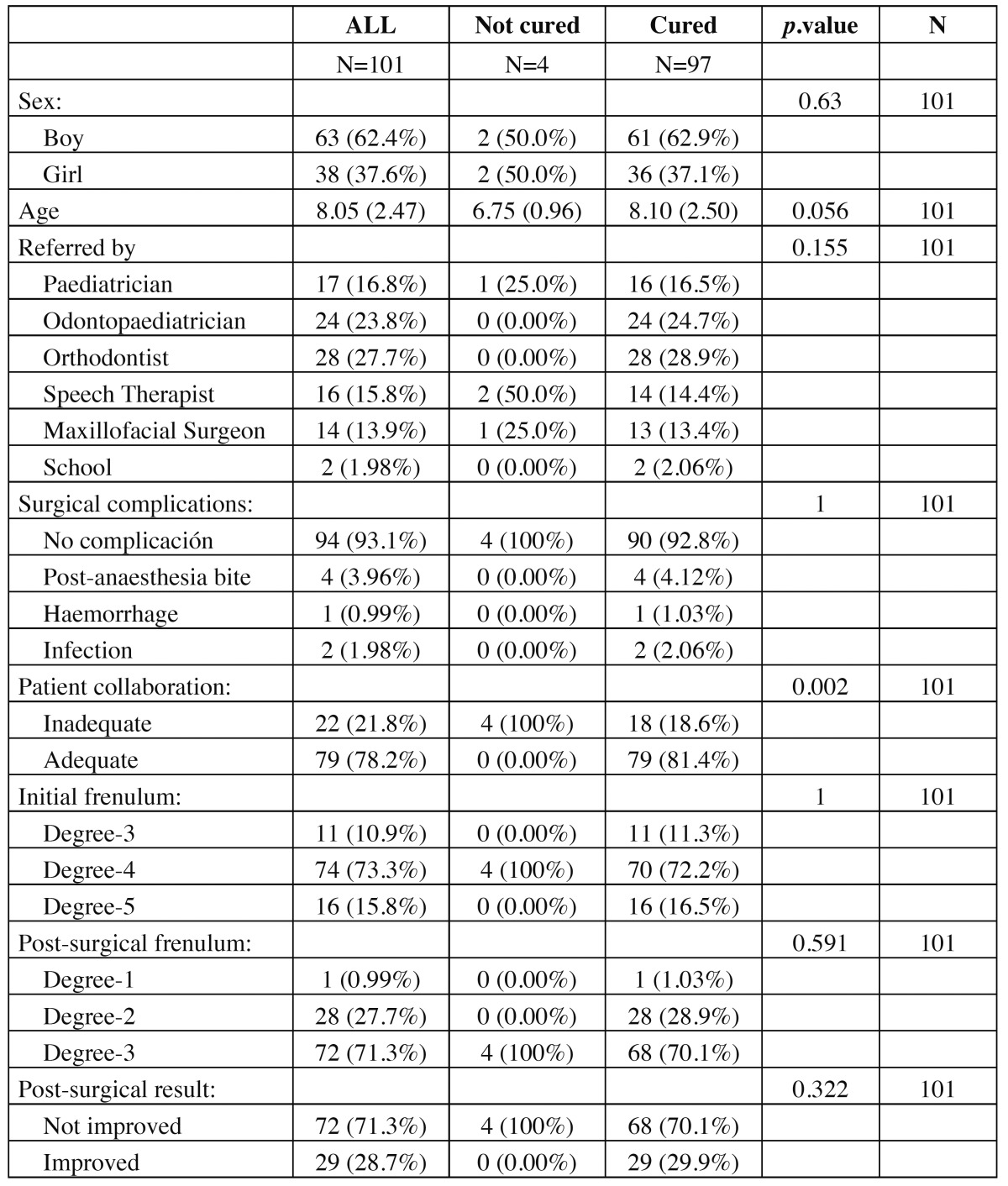
General description of the participants according to the degree of final Ankyloglossia.

In all patients the tongue is released after the lingual frenectomy and plasty; what means that the tongue tip can reach its highest point and has a totally free movement. However, the post surgical ankyloglossia grade is re-evaluated in the first rehabilitation session, in which some patients show moderate lingual mobility impairment. The results show that during this evaluation the degree of ankyloglossia has been improved, considering correction (degrees 1 or 2) in 29 (28%) of the patients (95% CI: 20%, 38%). In addition to this, when all the posts urgical orofacial rehabilitation sessions have been performed, the post surgical ankyloglossia grade is evaluated again, reaching a correction of 97 (96%) of the participants (95% CI: 90%, 98%).

There was some (postoperative) complication in 7 (6%) of the participants (95% CI: 2%, 13 %): 4 tongue bites, 1 hemorrhage and 2 infections, although none of these were serious ( Table 1). The collaboration of the patient in the undertaking of post-surgical exercises was considered adequate in 79 (78%) of those operated (95% CI: 68 %, 85 %).

In 4 of the cases, reducing the degree of ankyloglossia was not achieved after the surgical intervention and neither after the orofacial rehabilitation. In none of these 4 patients was their collaboration considered adequate, being their lack of it the only predictor which reached a statistical significance (*p*-value = 0.001). The average age of the patients who managed to be cured did not reach a statistical significance.

## Discussion

The tongue is an important oral structure which intervenes in speech, the position of the teeth, swallowing and certain social activities (16).

There is continuing controversy over the diagnostic criteria and treatment of ankyloglossia. (17) Currently, there is some disparity and controversy about the different classifications. On occasions, the need to quantify the degree of ankyloglossia with a mathematical formula in which different measurements have to be taken makes it difficult to reach the quorum for diagnosis among the professionals. (13-20) In other cases, only the degrees of frenula are considered for surgery, omitting the rest. Others lead to some confusion as regards defining the acuteness of the frenulum inversely to the numeration assigned to them (17-26).

The diagnosis of ankyloglossia of Cuestas *et al*., is based on anatomical criteria (inspection and palpation of the lingual frenulum) and functional ones (lifting, extension and lateralisation of the tongue) (16).

Haham *et al*., found no statistical correlation between the Coryllos type of lingual frenulum and the presence of breastfeeding difficulties (25).

The Hazelbaker Assessment Tool for Lingual Frenulum Function (HATLFF) has been developed to give a quantitative assessment of tongue-tie and recommendation about frenotomy (release of the frenulum) (27,28).

Five appearance items, such as the length of lingual frenulum (>1 cm, 1 cm, <1 cm) and seven functional items, such as extension of the tongue (tip over the lower lip, tip over lower gum only, neither) are assessed (28). Ballard *et al*., have explained how to score each item (29). Hazelbaker has demonstrated that the tool has content validity, however, it needs to be formally assessed for reliability (27,28,30).

Jamilian *et a*., evaluated the distance between the uppermost point of the lingual frenulum and its insertion into the oral floor. The subjects were categorised from having no ankyloglossia to severe tongue-tie based on the measurements (1).

Ankyloglossia can be classified into 4 classes based on Kotlow’s Classification as follows: Class I: Mild ankyloglossia (12 - 16 mm); Class II: Moderate ankyloglossia (8 - 12 mm), Class III: Severe ankyloglossia (4 - 8 mm), Class IV: Complete ankyloglossia (< 4 mm). Class III and IV tongue-tie categories should be given special consideration because they severely restrict the tongue’s movement (2).

Our criteria of surgical intervention for the degree of frenulum are the following: We consider frenula categorised as degree 4 or 5 require surgery, and the lingual frenulum degree 3 is only considered for surgery if is related to another alteration, pathology or disorder. In those doubtful cases, we establish a period between 3 and 6 months of rehabilitation before a frenectomy is considered. Furthermore, we assess the problems of speech, dental malocclusion, atypical swallowing, etc.

Our criteria of surgical intervention takes into account the maturation processes.

Mature deglutition: It is considered that when the first molars appear, the real chewing movements begin and the learning of a mature swallowing starts. Some authors affirm that the majority of the children manage it between 12 and 15 months. Other authors consider that it is at three years and others affirm that the process can be considered complete around 4 or 5 years old (4,10,13).

Evolutionary process of speech: It is called evolutionary dyslalia, that phase of development of infant language in which the child is not capable of repeating by imitation the words they hear, in order to form the acoustic-articulatory stereotypes correctly. Due to this, the words are repeated incorrectly from a phonetic point of view. Therefore, the symptoms which appear are those belonging to dyslalia, given the articulation difficulty. Within the normal development of the maturity of a child, these difficulties are gradually overcome and only if they remain beyond 4/5 years can they be considered pathological (13,23,30). Hence, we then establish that the appropriate age for surgery is around 4/5 years.

Most authors agree that the choice of treatment of ankyloglossia is the frenectomy and lingual plasty; the incision can be done with a cold scalpel, scissors, electrosurgical unit, or laser (16-22). Nowadays, several surgical techniques have been described to correct an abnormal frenulum. The following techniques are of particular interest in Pediatric Dentistry: frenotomy and frenectomy with the use of one haemostat, two haemostats, a groove dissector or laser (18,21,23).

Heller *et al*., recommend the Z-plasty since our data indicated that the 4-flap Z-frenuloplasty was superior to the horizontal to vertical frenuloplasty with respect to tongue lengthening, protrusion, and articulation improvement for patients with symptomatic ankyloglossia (22).

Choi *et al*., consider that the conventional surgery with Z-plasty is not effective and perform myotomies of the genioglossus muscles to free the muscles. The conventional method alone is not as effective; therefore, the authors studied the addition of a partial myotomy of the genioglossus muscle along with mucosal layer release for treatment. The combined application of conventional Z-plasty and genioglossus muscle release (20).

Sane *et al*., recommend that after surgery, speech therapy lessons for improvement of speech are required (21).

In the case that there are other functional disorders or speech alterations or both, the treatment is initiated for the normalisation of the function and the correction of the altered sounds once the patient is discharged from the post-surgical orofacial rehabilitation as a result of a frenectomy. With regard to speech, the most frequent alteration is the dyslalia of the /R/. Priority is given, when both alterations occur, the reason for the medical consultation. It is not advisable to treat both alterations parallelly.

## Conclusion

The treatment of choice for ankyloglossia is the frenectomy and lingual plasty associated to lingual myofunctional rehabilitation. Myofunctional rehabilitation begins one week before the surgical intervention, and the patient is explained the lingual praxis that will be carried out in the following weeks. The objective of this protocol is that the patient learns the exercises without pain. The results of our study demonstrate that the surgical technique of frenectomy with rhomboid plasty, the patient improves its lingual mobility. If this is reinforced with rehabilitation exercises and good patient collaboration, the results are excellent.
